# High Content Analysis Provides Mechanistic Insights on the Pathways of Toxicity Induced by Amine-Modified Polystyrene Nanoparticles

**DOI:** 10.1371/journal.pone.0108025

**Published:** 2014-09-19

**Authors:** Sergio Anguissola, David Garry, Anna Salvati, Peter J. O'Brien, Kenneth A. Dawson

**Affiliations:** 1 Centre for BioNano Interactions, School of Chemistry and Chemical Biology, University College Dublin, Belfield, Dublin, Ireland; 2 Pathology Department, School of Veterinary Science, University College Dublin, Belfield, Dublin, Ireland; National Institute of Health (NIH), United States of America

## Abstract

The fast-paced development of nanotechnology needs the support of effective safety testing. We have developed a screening platform measuring simultaneously several cellular parameters for exposure to various concentrations of nanoparticles (NPs). Cell lines representative of different organ cell types, including lung, endothelium, liver, kidney, macrophages, glia, and neuronal cells were exposed to 50 nm amine-modified polystyrene (PS-NH_2_) NPs previously reported to induce apoptosis and to 50 nm sulphonated and carboxyl-modified polystyrene NPs that were reported to be silent. All cell lines apart from Raw 264.7 executed apoptosis in response to PS-NH_2_ NPs, showing specific sequences of EC50 thresholds; lysosomal acidification was the most sensitive parameter. Loss of mitochondrial membrane potential and plasma membrane integrity measured by High Content Analysis resulted comparably sensitive to the equivalent OECD-recommended assays, allowing increased output. Analysis of the acidic compartments revealed good cerrelation between size/fluorescence intensity and dose of PS-NH_2_ NPs applied; moreover steatosis and phospholipidosis were observed, consistent with the lysosomal alterations revealed by Lysotracker green; similar responses were observed when comparing astrocytoma cells with primary astrocytes. We have established a platform providing mechanistic insights on the response to exposure to nanoparticles. Such platform holds great potential for *in vitro* screening of nanomaterials in highthroughput format.

## Introduction

The introduction of nanoparticle in numerous industrial [1], [2] and biomedical applications [3], as well as consumer products [4], [5] has raised concerns in relation to their impact on the environment and human health [6], [7]. To promote the safe and responsible application of new and existing materials in the growing nanotechnology era it is required that engineered nanomaterials are assessed for their impact on the environment and human health. A more detailed understanding of how nanoparticles interact with biological systems is required in order to understand how nanoparticle exposure will affect individuals both in an acute and chronic exposure scenarios. The current of understanding is due to the diversity of nanomaterials chemical composition, size distribution, total surface area, surface charge and other physico-chemical characteristics which can lead to multiple and diverse interactions with the surrounding environment and with biological systems [8], [9].

When particles reach a size in the nanometre range they develop new properties due to their increased volume to surface area ratio, resulting in increased surface energy; this phenomenon completely alters the nanomaterial properties when compared to their larger bulk form [10] and this can be exploited for various applications that span from industry to consumer products. Nanomaterials' small dimensions allow them to enter the body (mainly by ingestion and inhalation) and potentially gain access to blood stream and become systemic in the body [11]. Once nanomaterials gain systemic access, they can accumulate in internal organs of the body; experimental evidence in animal models has shown accumulation mainly in the liver and kidneys [12], [13] and it is still very debated whether NPs also can cross the Blood Brain Barrier and access the brain [14], [15]. These properties make NPs very promising for biomedical applications such as drug delivery. When nanoparticles are suspended in biological fluids, in order lower their surface energy, they adsorb proteins and other biomolecules from the surrounding environment, forming a layer called “corona”[16]–[19]. It is believed that this layer defines the biological identity of the NPs and affects nanoparticle-cell interactions. Nanomaterials are taken up by cells through active, energy-dependent endocytic pathways and in many cases they are transported to the lysosomes [20]–[22]. Once in the lysosomes, experimental evidence shows that NPs in manny cases are not exported and accumulate into lysosomes without any evident damage, as cells continue to divide [23]. In other cases some NPs are known to be toxic to cells. For instance cationic PS-NH_2_ NPs have been described to induce cytotoxicity by caspase mediated apoptotic pathways at relatively low concentrations [24]–[27]. Once cells undergo apoptosis pro-apoptotic Bcl-2 family proteins assemble on the mitochondrial membrane and open pores that release apoptogenic factors responsible for activation of the caspase cascade. This caspase cascade leads to controlled cell death via apoptosis [28]. The relationship between nanoparticle surface properties and their potential toxicity are largely unknown; moreover little is known about the molecular mechanisms governing nanoparticle cytotoxicity. High Content Analysis (HCA) has already been successfully used in the field of drug discovery [29]–[32] and toxicology [33]–[35] for the ability to analyse numerous samples in the same experiment. Recently HCA has also been suggested as a powerful technology to assess potential toxicity of nanomaterials [36]–[39].

In this work we developed a multi parametric *in vitro* platform to assess potential cyctoxicity induced by nanoparticles using High Content Analysis (HCA). The fluorescent microscopy HCA cytotoxicity platform employs fluorescent dyes with complementary excitation/emission spectra to examine: changes in nuclear morphology, mitochondrial membrane potential, cytosolic calcium levels, acidificaton of the lysosomes and plasma membrane integrity. This versatile multi-parametric platform enables a user to analyse multiple parameters for a high number of samples, minimizing input while maximizing the experimental output.

In order to assess potential toxicity of nanomaterials to mammalian cells, we decided to use cell lines where nanoparticle uptake was previously documented. As our HCA platform was developed as an *in vitro* approach to mimic human exposure to nanoparticles we used cell lines representative of many organs, focussed on inhalation scenarios and clearance. We believe that the development of a High Content multi-parametric *in vitro* cell toxicity platform will change the efficiency of nanoparticle safety testing by combining the output of several traditional assays and still complying with OECD regulations. The existing HCA platfom could be implemented to become a tool to assess exposure of specific tissues and organ-like structures for a realistic risk assessment of nanomaterials providing insights on the mechanisms of toxicity and establishing the link between molecular pathways of toxicity and nanomaterials surface properties; further, application of HCA would open the way to develop system biology approaches that are predictive of nanomaterial impact on human health.

In order to establish this platform we used PS-NH_2_ NPs as candidate positive control for NPs-induced apoptosis. This allowed us to confirm by HCA the same scenario previously proposed [27], [40] and provide new insights on the cellular pathways activated.

## Materials and Methods

### Nanoparticles and nanoparticles dispersions

Commercial Nanoparticles used were 50 nm sigma blu amine-modified polystyrene (PS-NH_2_) (Sigma Aldrich), 50 nm sulphonated polystyrene (PS-Plain) (PolySciences) and 50 nm Carboxyl-modified polystyrene (PS-COOH) (Polysciences). All the commercial NP dispersions were stored at 4°C, and used as provided, without performing any cleaning step or other manipulation, within 1 year of arrival. Additionally 50 nm Silicon Dioxide (Polysciences), 180 nm Cerium Dioxide (Umicore), 55 nm Titanium Dioxide (Vive Science) [41] and 100 nm Zinc Oxide (Vive Science) NPs were used for descriptive purposes. All experiments were performed using the same batch of nanoparticles. Physico-chemical properties of NPs were characterised by Dynamic Light Scattering (DLS) and zeta potential measurements at 25°C once the commercial dispersions were diluted in reference buffers, including 10 mM NaCl (Sigma Aldrich), PBS (Sigma Aldrich); The NP dispersion was also characterised in complete cell culture medium DMEM glutamax (Life Technologies) supplemented with 10% Fetal Bovine Serum (Life Technologies) after incubation for 24 hours and 72 hours at 37°C in order to describe NPs properties in the conditions applied fo *in vitro* testing. NP dispersions were diluted to the highest exposed dose to the cell cultures (100 µg/ml) and size distribution, poly dispersity index and zeta potential were recorded. Data are shown as mean +/− SD of triplicate measurements from three independent experiments.

#### Reagents

1321N1 human astrocytoma, SHSY5Y human neuroblastoma, HepG2 human hepatocellular carcinoma, HEK 293 Human Embryonic Kidney and A549 human alveolar adenocarcinoma cells were purchased from ATCC; NHA human primary astrocytes were purchased from Lonza; Raw 264.7 Murine macrophage were purchased from ECACC; hMECD immortalised human microvascular endothelial capillary cells were kindly donated by Florence Miller, B.B. Wecksler (Inserm, France).

The fluorescent dyes, Hoechst 33342, TMRM, Fluo-4, Lysotracker Green and TOPRO-3, were purchased from Life Technologies.

All chemicals were purchased from Sigma Aldrich.

#### Cell culture

SHSY5Y, HepG2, Raw 264.7, and HEK 293 cells were cultured in DMEM glutamax (life technologies) supplemented with 10% FBS (Life technologies); 1321n1 cells were cultured in DMEM glutamax (life technologies) supplemented with heat-inactivated 10% FBS (Life technologies). A549 cells were cultured in MEM (Life Technologies) supplemented with 1% glutamine 10% FBS (Life technologies); hCMEC/D3 cells were cultured in EBM-2 medium supplemented with vascular endothelial growth factor (VEGF), insulin-like growth factor-1 (IL-1), epidermal growth factor (EGF), basic fibroblast growth factor (bFGF), fetal calf serum (2%), gentamicin sulphate/amphotericin B and hydrocortisone (Lonza Biosciences); NHA cells were cultured in ABM Astrocyte Basal Medium supplemented with Rh EGF, insulin, ascorbic acid, GA-1000, L-glutamine and 10% heat-inactivated FBS according to manufacturer's instructions (Lonza Clonetics Astrocyte Cell Systems) and 1% Penicillin/streptomycin; cells were grown at 37°C in a humidified atmosphere of 5% CO_2_/95% air. Cells were routinely sub-cultured three times a week using trypsin (0.25%) (Life Technologies). Serum heat inactivation was performed by incubating the serum at 56cyte Cell Systems).

#### High Content Analysis

High Content Analysis (HCA), an automated epifluorescence microscopy approach with proprietary acquisition/analysis software was used to assess cellular cytotoxicity in a multiparameter approach. HCA was performed using a modified version of the approach established by Prof. O'Brien [33].

Briefly, 5×10^3^ cells were seeded in a clear flat bottom 96 well plate (Cell Star) in 100 µl of cell culture medium containing 10% FBS. After 24 hours nanoparticle dispersions were prepared as 3× the final concentration required in cell culture medium containing 10% FBS, then 50 µl of particle suspensions were added to the cell-containing wells to reach a 1× concentration. Equivalent volume of water to the highest volume of nanoparticles was applied as vehicle in all experiments. The dispersants from the nanoparticle suspensions was tested at the same volume which did not induce any alteration of the parameters analysed (data not shown). Cells were incubated with nanoparticles at a final concentrations of 0.3 µg/ml, 0.7 µg/ml, 1.5 µg/ml, 3 µg/ml, 6 µg/ml, 12 µg/ml, 25 µg/ml, 50 µg/ml and 100 µg/ml, for 24 or 72 hours.

At the end of the incubation period 50 µl of medium was removed from the wells and replaced with 50 µl of a 3× concentrated solution containing the following dyes: Hoechst (400 nM) to identify cell nuclei and assess changes in nuclear morphology, TMRM (20 nM) to assess loss of mitochondrial membrane potential, Fluo-4 (1 µM) to measure increase in cytosolic calcium, Lysotracker green (200 nM) to assess lysosomal acidification (assessed in a separate experiment), TOPRO-3 (800 nM) to evaluate plasma membrane permeabilization. After 1 hour incubation, cells were analysed by High Content Analysis using the Arrayscan VTI 740 (Thermo Scientific). Images were acquired using a 20× objective and fluorescence intensities were collected using the following combination of excitation/emission filters: Hoechst was excited through a 365+/−50 nm band pass filter and fluorescence emission was collected through a 515+/−20 nm band pass filter; TMRM was excited through a 549+/−8 nm band pass filter and fluorescence emission was collected through a 600+/−25 nm band pass filter; Fluo-4 or Lysotracker green were excited through a 475+/−40 nm band pass filter and fluorescence emission was collected through a 515+/−20 nm band pass filter; TOPRO-3 was excited through a 655+/−30 nm band pass filter and fluorescence emission was collected through a 730+/−50 nm band pass filter. Image analysis was performed using the cell health profile bioapplication. Data acquisition was configured to image an average of 300 to 500 cells within 10 acquired images (fields). Analysis parameters were set according to manufacturer's instructions and fixed thresholds were used for each parameter to separate background noise from fluorescence. Dr. Chandrasekaran at Thermo Scientific USA implemented a custom made algorithm so that the Cell Health Profile bioapplication reported field view details (average intensity per cell using a fixed threshold in each field acquired) for the following parameters: cell numbers, nuclear size, nuclear intensity, TMRM intensity, Fluo-4 intensity, Lysotracker green intensity and TOPRO-3 intensity.

Data was exported to Prism where the EC_50_ and IC_50_ were calculated by fitting the data with a sigmoid curve. Data are shown as mean +/− SD of 45 fields acquired from three independent experiments performed in triplicate.

The detailed properties of Lysotracker green positive lysosomes were analysed using the Spot Detection Bioapplication. Lysotracker positive objects were identified and separated using a 3 sigma algorithm. Cell details were acquired and they were shown as mean +/− SD of a representative experiment performed in triplicate.

#### Cell death assessment by Flow Cytometry

Cell membrane permeability was measured by Propidium Iodide uptake in cells, as sign of cell damage. 1×10^5^ cells were seeded in a 24-well plates, and incubated for 24 hours prior to addition of nanoparticles. After 24 hours the medium was replaced with medium containing NPs diluted from the commercial dispersion in cell culture medium containing 10% FBS to the concentrations indicated previously (1.5 µg/ml, 12 µg/ml, 25 µg/ml, 50 µg/ml and 100 µg/ml). Cells were incubated with NPs at 37°C with 5% CO_2_ for 24 hours. Samples were washed with PBS and harvested with 0.25% trypsin. Cells were resuspended in PBS containing 5 µg/ml Propidium Iodide. The samples were then incubated on a thermal mixer shaking at 600 rpm for 15 minutes at 37°C.

Samples were acquired on a C6 flow cytometer (BD Accuri) equipped with a 20 mW 488 nm laser. Fluorescence emission of Propidium Iodide was recorded through a 585+/−40 nm long pass emission filter. The percentage of PI-positive cells was reported as mean +/− SD of three independent experiments performed in triplicate.

#### MTS cell viability assay

Cellular viability was determined by the MTS assay, which measures the reduction of 3-(4,5-dimethylthiazol-2-yl)-5-(3-carboxymethoxyphenyl)-2-(4-sulfophenyl)-2H-tetrazolium (MTS) to formazan by mitochondria in viable cells. 1.5×10^4^ cells were seeded in each well of a 96-well plate (Cell Star) and grown in a humidified incubator at 37°C with 5% CO_2_ for 24 hours. The nanomaterials to be tested were diluted from the commercial dispersion in cell culture medium containing 10% FBS and added to the wells at the doses indicated previously (1.5 µg/ml, 12 µg/ml, 25 µg/ml, 50 µg/ml and 100 µg/ml). After the incubation periods the cell culture media was removed from each well. The CellTiter 96 AQueous One Solution Reagent (Promega) was added to each well of the 96 well plate. The plate was incubated with the MTS reagent for 60 minutes at 37°C in a humidified, 5% CO_2_ atmosphere. Formazan absorbance was measured at 490 nm using a microtiter plate reader (Varioskan Flash, Thermo Scientific, MA, USA). Recorded data were processed as follows: the average background level was subtracted from each well. The resulting absorbance from each dosing well was normalized to the absorbance value of untreated cells. After normalization, the values from a single dose in the technical replicates were averaged and standard deviation was determined. Additional control experiments, namely the MTS reagent mixed with nanoparticles only, were performed to assess potential interference of the nanoparticles, and did not reveal any interference of the NPs used with the MTS assay.

Data was reported as mean +/− SD of three independent experiments performed in triplicate.

Data was exported to Prism to assess statistical significance.

### Assessment of lipid intracellular accumulation

The lipidTOX phospholipidosis and steatosis detection kit for High Content Screening (Life Technologies) was used to assess accumulation of phosphor- and neutral lipids inside cells according to manufacturer's instructions.

Briefly 5×10^3^ cells were seeded into each well of a 96 well plate and exposed to PS-NH2 and PS-COOH NPs for 24 and 48 hours as previously indicated; the LipidTOX Red phospholipidosis detection reagent was applied at the same time of NP exposure.

After exposure cells were fixed with 4% formalin and stained with Hoechst 33342 and the LipidTOX Green neutral lipid stain. Fluorescence intensity was acquired by using HCA as previously described.

### Statistical analysis

HCA data were exported to Prism; IC50/EC50 values were calculated by fitting a sigmoid curve (log (inhibitior) v.s. normalised response – variable slope or log (actuator) v.s. normalised response – variable slope respectively) in.

Statistical analysis of flow cytometry data was performed using one way Anova. Samples were considered significantly different from control when the p value was lower than 0.05 (95% confidence).

## Results

### Establishment of a HCA platform to identify the cellular pathways of toxicity

In order to establish a High Content Analysis in vitro platform to assess the potential impact of nanomaterials for human health and identify the cellular pathways responsible; we selected a panel of cell lines as representative cells from the main routes of exposure, accumulation and clearance of NPs from the body. A549 alveolar adenocarcinoma cells were selected to mimic the inhalation route, hCMEC/D3 endothelial cells and Raw 264.7 macrophages described the interaction of NPs with the immune system and endothelium that forms the blood vessels respectively; HepG2 hepatocellular carcinoma cells were used to study eventual effects to the liver and 1321n1 astrocytoma, and SHSY5Y neuroblastoma cells were chosen to monitor potential neurotoxic effects. Finally HEK293 embryonic kidney cells were selected to assess the potential impact on the kidneys as the main clearance route (Fig. S1 in File S1).

### Polystyrene NPs are well dispersed in the complete cell culture medium over the duration of the exposure to cells

**Figure 1:**
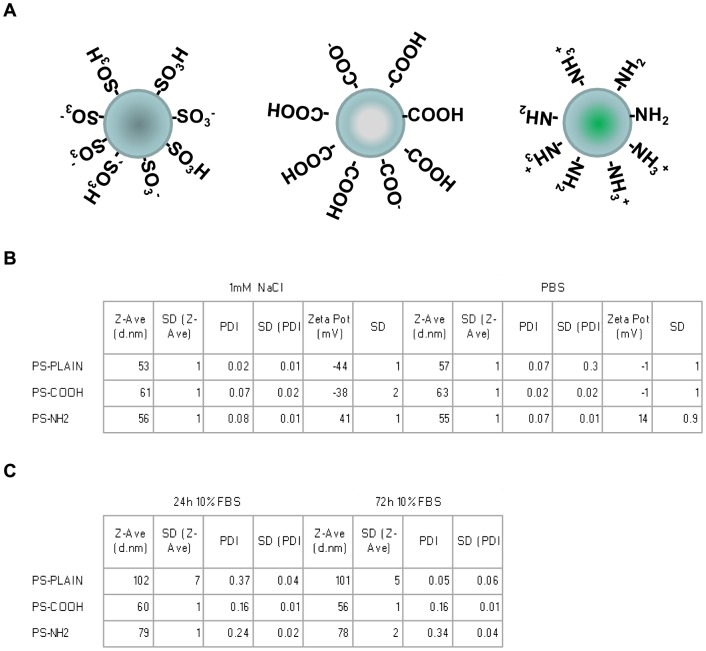
PS-NH_2_, PS-COOH and PS-plain NPs preserve the dispersion characteristics when suspended in complete cell culture medium. The NPs employed, PS-NH_2_, PS-COOH and PS-Plain have a narrow size distribution and similar hydrodynamic size. A. Cartoon shows schematically the surface modifications of the Polystyrene NPs used. B. DLS data acquired for PS-NH_2_, PS-Plain and PS-COOH NPs incubated in 1 mM NaCl and PBS for 24 hours and 72 hours. Hydronamic size (Z-Ave), Polydispersity Index (PDI) and Zeta potential were measured. The NPs showed similar dispersion properties. C. DLS data acquired for PS-NH_2_, PS-Plain and PS-COOH NPs incubated in complete cell culture medium for 24 hours and 72 hours. Hydronamic size (Z-Ave) and Polydispersity Index (PDI) were measured. PS-COOH and PS-Plain NPs retained similar dispersion properties to the original dispersion while PS-NH_2_ NPs showed slight aggregation. Data are shown as average +/− SD of 3 experiments repeated in triplicate.

In order to establish the HCA screening platform we initially used nanoparticles that have been previously characterised in our group; amine-modified polystyrene 50 nm (PS-NH_2_) NPs induced apoptosis in astrocytoma 1321N1 cells at concentrations between 50–100 µg/ml [27], [40], while carboxyl-modified (PS-COOH) or sulphonated polystyrene (PS-Plain) 50 nm NPs did not elicit any biological effect at the same concentrations. A schematic representation of the selected NPs is illustrated in Fig. 1A. We therefore performed dose response experiments at 24 hours and at 72 hours to mimic acute and chrnoic response in the cell models selected. Before the nanoparticles were exposed to the selected cell lines we verified the dispersion characteristics of the NPs utilized. PS-NH_2_ and PS-COOH NPs have been extensively characterised and have shown to be very stable once dispersed in cell culture medium, showing negligible agglomeration [42]–[44]. NPs physico-chemical properties were assessed by Dynamic Light Scattering (DLS) after diluting the commercial dispersion in reference buffers (10 mM NaCl and PBS) as well as the complete cell culture medium supplemented with 10% fetal bovine serum (FBS) used to grow the cell cultures, in order to assess whether the effective dose presented to the cells was consistent with the applied dose. The DLS size measurements showed that when dispersed in PBS the average hydrodynamic size of the three NPs was close to the nominal size indicated by the manufacturer; the poly dispersity index (PDI) was in a range comparable with a monodispersed suspension, suggesting that the nanoparticles are adequately dispersed in the reference buffers; zeta potential was negative for PS-Plain (−0.2 mV) and PS-COOH (−0.3 mV), while the amine-modified PS-NH_2_ had a highly positive charge (+14 mV) (Fig. 1B). The DLS measurements in the cell culture medium showed very similar average hydrodynamic size and a small increase in PDI compared to the reference buffers, indicating that the nanoparticles used are still reasonably mono-dispersed in the cell culture medium (Fig. 1C). Further analysis showed that while the average size of the dispersion increased once nanoparticles were dispersed in cell culture medium the dispersions remained stable over the duration of exposure to cells with negligible signs of agglomeration (Figure S2 in File S1). The zeta potential of the three nanoparticles isolated after incubation with FBS was previously measured, showing that the protein corona formation on the nanoparticle surface neutralized the surface charge resulting in a zeta potential close to zero [17], [27].

### HCA resolves apoptotic and necrotic cell death responses in the model cell lines investigated

**Figure 2:**
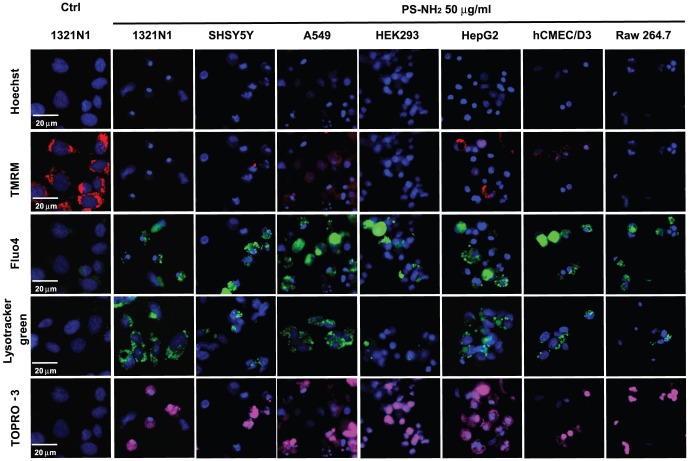
Representative images of the cell lines exposed to PS-NH_2_ NPs showed alteration of the cellular parameters investigated associated with cell death. The indicated cell lines were exposed to vehicle (ctrl) or increasing concentrations of PS plain, PS-COOH or PS-NH_2_ NPs for 24 hours; images were acquired using HCA to assess changes in nuclear morphology (Hoechst), mitochondrial membrane potential (TMRM), cytosolic calcium levels (Fluo-4), lysosomal acidification (Lysotracker green), and plasma membrane integrity (TOPRO-3); representative images of 1321N1 cells exposed to vehicle (ctrl), and 1321n1, SHSY5Y, A549, Raw264.7, hCMEC/D3, HepG2, HEK293 cells exposed to PS-NH_2_ (100 µg/ml) revealed changes in fluorescence intensity of the fluorescent dyes used, indicating that PS-NH_2_ NPs caused alteration in all the parameters analysed. Scale bar = 20 µm.

**Figure 3:**
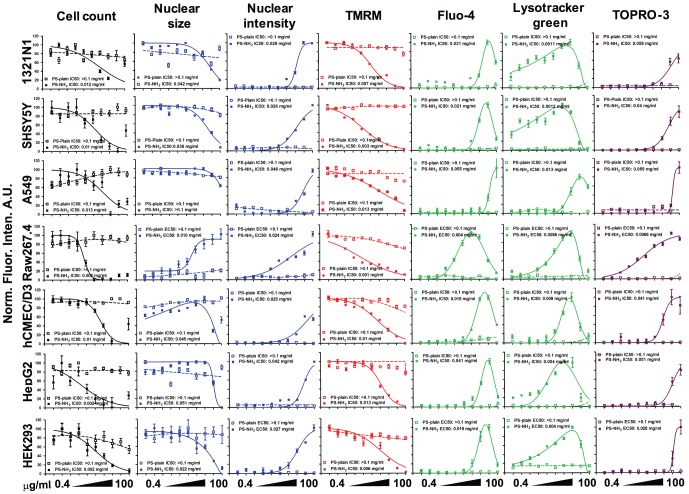
Exposure to PS-NH_2_ NPs for 24 hours caused alteration of all the parameters resulting in cell death while PS-COOH or PS-Plain NPs did not exert any effect. The indicated cell lines exposed to vehicle (ctrl) or increasing concentrations of PS plain or PS-NH_2_ NPs for 24 hours were acquired using HCA to assess changes in nuclear morphology (Hoechst), mitochondrial membrane potential (TMRM), cytosolic calcium levels (Fluo-4), lysosomal acidification (Lysotracker green), and plasma membrane integrity (TOPRO-3); The graphs show for each cell line and each parameter the dose dependent change caused by PS-NH_2_ NPs (continuous line) while PS-COOH NPs did not cause any effect (dashed line). Dose responses for PS-Plain NPs were not shown as they overlapped with PS-COOH dose responses. Data are shown as average +/− SD of 45 acquired images from three independent experiments. Curve fitting and EC50/IC50 were calculated as described in the Methods. Fluo-4 and Lysotracker green dose response plots were fitted with a gaussian curve for visual purposes only.

Once assessed that the nanoparticles to be used were reasonably mono-dispersed in the complete cell culture medium we proceeded to perform dose response experiments using a multi-parametric approach that measures changes in cell numbers, nuclear size and intensity, mitochondrial membrane potential, cytosolic calcium, acidification of the lysosomes and plasma membrane integrity as indicated in the materials and methods section. The panel of selected cell lines was exposed to increasing concentrations (0.4 µg/ml, 0.75 µg/ml, 1.5 µg/ml, 3 µg/ml, 6 µg/ml, 12.5 µg/ml, 25 µg/ml, 50 µg/ml and 100 µg/ml) of PS-NH_2_, PS-Plain and PS-COOH NPs for 24 and 72 hours; after 24 hours exposure all the selected cell lines displayed changes in the measured parameters in a dose-dependent fashion; representative images show how, compared to control cells, a reduction in nuclear size, TMRM fluorescence, and increase in nuclear fluorescence, fluo-4, lysotracker green and TOPRO-3 were observed for all cell lines exposed to 50 µg/ml of PS-NH_2_ NPs, except for Raw264.7, where increase in nuclear size was observed instead (Fig. 2). Control images for the full panel of cells investigated are included in the Figure S3 in File S1. This difference in nuclear morphology suggests that a different signalling pathway could be responsible for cell death in Raw 264.7 cells. By performing the image analysis as described in the Methods we generated dose response profiles for all the parameters analysed at 24 hour and 72 hours. An example of the results forPS-NH_2_ and PS-Plain NPs is shown in Fig. 3 (data for PS-Plain NPs were comparable to PS-COOH NPs). PS-COOH and PS-Plain NPs did not cause any alteration in the cellular markers compared to cells exposed to vehicle, indicating that they did not elicit any cytotoxic response; however, PS-NH_2_ NPs showed dose dependent alteration of all the measured parameters resulting in acute toxicity for all the cell lines investigated (Fig. 3).

Interestingly, for all the cell lines except Raw264.7, the decrease in nuclear size and increase in nuclear intensity, indicative of nuclear condensation, associated with mitochondrial depolarisation (TMRM), increase in lysosomal acidification (Lysotracker green), calcium deregulation (Fluo-4) and plasma membrane permeabilization (TOPRO-3); this suggested that the cells underwent apoptotic cell death, consistently with previous results [26], [27], [40], and lysosomal damage; the same parameters indicated nuclear swelling after exposure of PS-NH_2_ NPs to RAW264.7 cells, strengthening the hypothesis that RAW264.7 cells underwent cell death through a different mechanism, potentially necrosis. Flow cytometric analysis of phosphatydilserine exposure measured by AnnexinV-FITC and Propidium Iodide (PI) uptake revealed a population of early apoptotic cells (AnnexinV positive and PI negative), following exposure of A549 cells, while only necrotic cells (AnnexinV positive and PI positive) could be observed following exposure of RAW264.7 cells, consistent with the previous findings showing that PS-NH_2_ NPs induced apoptosis in A549 cells [43], [45], and further supporting the hypothesis that RAW264.7 cells underwent necrosis as opposed to apoptosis (Figure S4 in File S1).

### Analysis of EC_50_ and IC_50_ reveals a sequence of thresholds consistent with the time sequence of apoptosis progression

**Figure 4:**
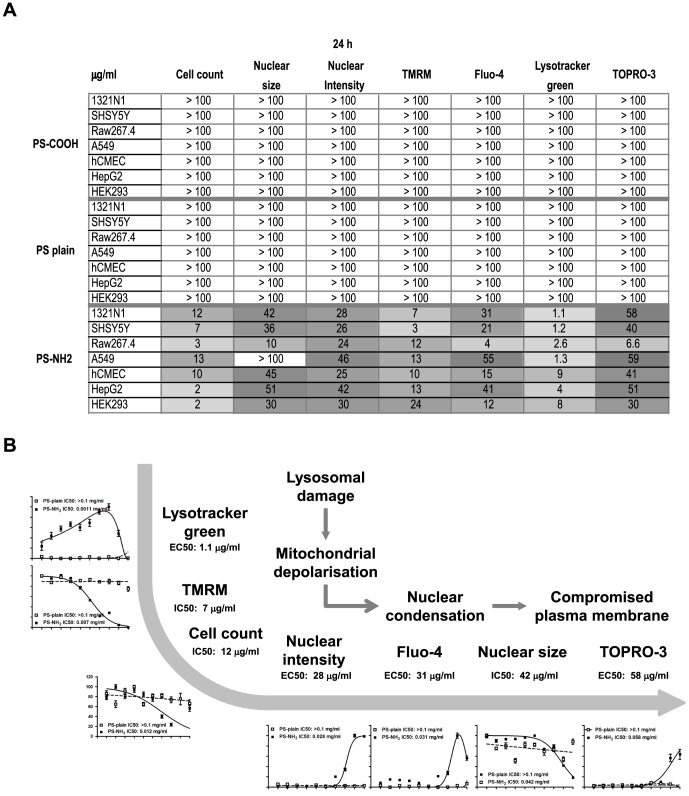
Sequences of EC50 thresholds suggest that all cell lines tested except Raw264.7 execute apoptotic cell death, while Raw264.7 undergo a non-regulated form of cell death. EC_50_/IC_50_ for each parameter measured for the selected cell lines exposed to vehicle (ctrl) or increasing doses of PS-Plain, PS-COOH or PS-NH_2_ NPs, for 24 hours or 72 hours were calculated as described in the Methods. EC50/IC50 measured values after exposure for 24 hours (A). C. Schematic representation showing EC50/IC50 threshold sequences as predictive of time sequences. The graphs and sequences of EC50 shown were derived from the measurements obtained for 1321n1 cells. After 24 hours exposure the sequence of thresholds suggests that all cell lines except RAW264.7 underwent apoptosis following initial lysosomal damage; the EC50 thresholds observed for RAW264.7 suggest a sudden/non regulated mechanism of cell death compatible with necrosis. The EC50/IC50 values for all the parameters analysed shifted to lower concentrations after exposure for 72 hours, suggesting that longer exposure can lead to potential toxicity for doses that resulted non toxic after 24 hour exposure.

A very valuable parameter used in toxicology to describe and normalize the response of cultured cells to different agents with variable degree of toxicity is the IC_50_/EC_50_ value. This was calculated from the average field fluorescence values for all the parameters analysed as described in the Methods section. The indicated EC_50_/IC_50_ values for PS-Plain and PS-COOH were all above 100 µg/ml, indicating that no effects were observed across all the concentrations tested. The most sensitive parameter for PS-NH_2_ nanoparticles (i.e the parameter with lowest EC_50_/IC_50_) observed for all cell lines except Raw264.7 was Lysotracker Green, followed by, in most of the cell lines investigted, mitochondrial depolarization, nuclear condensation, nuclear intensity increase, cytosolic calcium and then loss of plasma membrane integrity, consistent with apoptotic cell death as previously reported [27], [40]. On the contrary, the EC_50_/IC_50_ values for the measured parameters in Raw264.7 cells were very similar (Fig. 4A), suggesting that in this cell line these events happened simultaneously, and led to swelling of the nucleus, rather than its condensation, which is consistent with a necrotic type of cell death. The EC50/IC50 values measured after 72 hours of exposure revealed a similar sequence of thresholds, though shifted to lower concentrations (Fig. S5), supporting the cell death pathways identified, i.e. apoptosis v.s. necrosis.

This sequence of EC_50_ confirmed, in agreement with previous observations [27], that after nanoparticles were actively taken up by cells through the endocytic pathways and accumulated in the lysosomes, they activated an apoptotic process that led to depolarization of the mitochondria with subsequent release of apoptogenic factors, and activation of the caspase cascade, finally resulting in nuclear fragmentation and condensation and subsequently plasma membrane rupture (Fig. 4B). The sequence of EC50 thresholds shown was derived by the measurements from 1321n1 cells, and applies to the majority of cell lines investigated, which executed apoptosis. We could observe that in few cases some parameters did not follow the sequence strictly (e.g. Fluo4, cell counts and TMRM for HEK293 cells), nevertheless all cell lines undergoing apoptosis followed the recognised steps of the apoptotic cascade: lysosomal damage followed by mitochondrial impairment, nuclear condensation and loss of plasma membrane polarity. This imaging approach is suitable for the analysis of a broad range of nanomterials, examples of which can be found in Figure S6 of File S1. Further it can be easily translated to applications requiring fixed cells; as an example, intensity of cell nuclei stained with Hoechst 33342 measured in live and fixed cells was highly comparable Figure S7 in File S1).

### High Content Analysis proved to be as sensitive as traditional toxicology assays

The Organization for Economic Co-operation and Development (OECD) has compiled a list of recommended *in vitro* and *in vivo* assays to test for toxicity induced by chemicals [46]; these tests are accepted by the scientific and regulatory community for the assessment of industry and consumer products being released on the market; the same are currently tested for their applicability to nanomaterials, and have been adjusted when nanomaterials show interference. With numerous new nanomaterials being introduced in industrial and consumer products these OECD recommended assays might not provide sufficient output; for this reason we decided to compare two parameters assessed by our HCA screening platform with the respective OECD recommended in vitro assays, to assess whether HCA provided similar sensitivity with the advantage to measure multiple parameters and multiple nanomaterials in the same experiment. Mitochondrial activity measured by TMRM was compared with the MTS assay while uptake of TOPRO-3 resulting from loss of plasma membrane integrity was compared to uptake of PI measured by flow cytometry.

**Figure 5:**
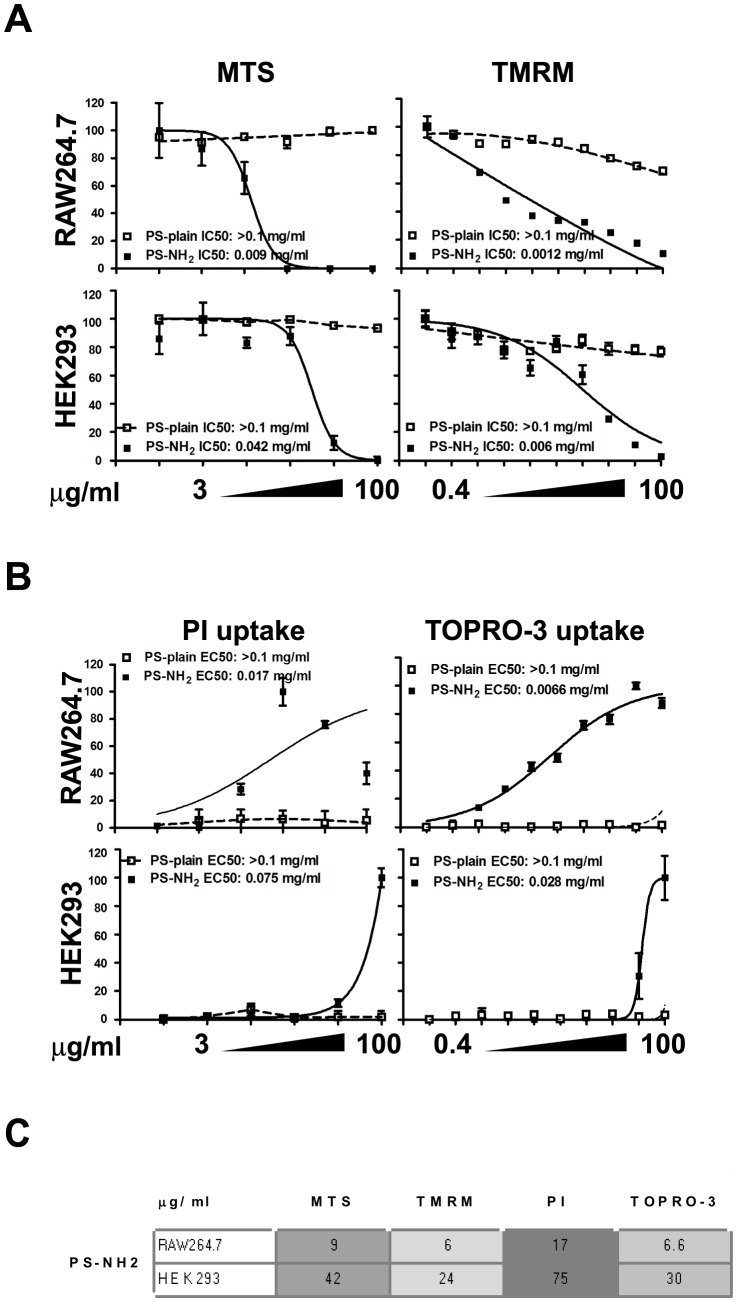
High Content Analysis shows comparable sensitivity to established cell viability assays. HEK293 and RAW264.7 cells were exposed to vehicle (ctrl) or increasing concentrations of PS-COOH or PS-NH_2_ for 24 hours; Cellular response was evaluated by HCA, MTS or PI uptake. A. Comparison of mitochondrial activity as measured by MTS and TMRM. B. Comparison of plasma membrane permeabilization using uptake of PI or TOPRO-3. C. EC50/IC50 thresholds and grey scale conditional formatting to highlight low and high thresholds. Cell imaging using TMRM and TOPRO-3 resulted as sensitive as MTS or flow cytometric analysis of PI uptake. HCA Data are shown as average +/− SD of 45 acquired images from three independent experiments. MTS and PI uptake data are shown as average +/− SD of thee independent experiments performed in triplicate. Curve fitting and EC50/IC50 were calculated using Prism.

Although the MTS cell viability assay and the TMRM fluorophore monitor different aspects of mitochondrial functionality, i.e the reductase enzymatic activity and the polarization of the mitochondrial membrane respectively, they were compared as both are dependent on healthy, functional mitochondria. RAW 264.7 and HEK 293 cells were exposed to positively charged PS-NH_2_ NPs and to PS-Plain NPs for 24 h and mitochondrial health was assessed using MTS cell viability assay and fluorescent microscopy HCA TMRM staining (Fig. 5A).

In each cell line, a reduction in mitochondria activity was observed with increasing concentration of PS-NH_2_ NPs. Mitochondria activity was unaffected by PS-Plain. By comparing the dose response to PS-NH_2_ NPs in both assays we observed a similar reduction in mitochondrial function, and the IC50 value showed that the HCA measurement of TMRM was as sensitive as the MTS assay (Fig. 5C). The flow cytometric PI uptake assay showed that PS-NH_2_ NPs caused cell death in a concentration dependent manner while the PS-Plain NPs did not cause increase in PI fluorescence at all tested concentrations. Comparable results were observed with our HCA assay (Fig. 5B), and also in this case the HCA assessment of cell death was as sensitive as the flow cytometric PI uptake assessment (Fig. 5C).

### High Content Analysis correlates lysosomal properties with cell death induced by PS-NH_2_ NPs

**Figure 6:**
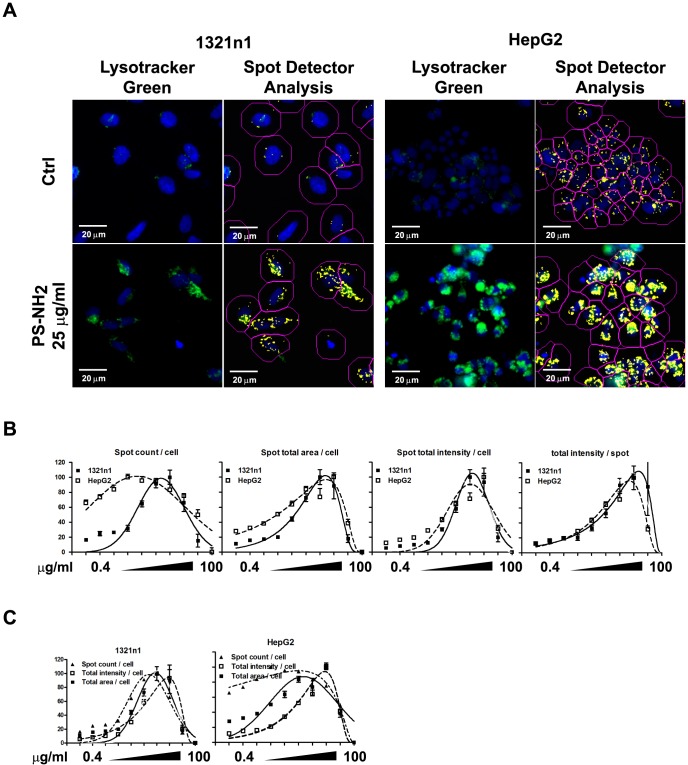
PS-NH_2_ NPs cause dose-dependent changes in lysosomal properties. I321N1 and HepG2 cells were exposed to vehicle (ctrl) or increasing concentrations of PS-COOH or PS-NH_2_ NPs for 24 hours and analysed by HCA. The Spot Detection Bioapplication was used to analyse the Lysotracker green positive vesicles inside the exposed cells. A. Representative images of cells exposed to vehicle or 25 µg/ml PS-NH_2_ NPs showing the increased Lysotracker green fluorescence and the analysis performed using the Spot Detector Bioapplication. Scale bar = 20 µm. B. Dose-dependent changes parameters associated with Lysotracker green positive vesicles: counts, area and intensity of Lysotracker green positive vescicles for each cell were calculated; dose dependent changes in individual spots were also recorded. C. Comparison among spot counts, spot total area and spot total intensity/cell within each cell line. The results suggest that increasing concentrations of PS-NH_2_ NPs cause swelling of lysosomes, as indicated by increased Lysotracker green intensity, followed by a reduction in spot counts and reduciton in the spot area/cell and their intensity, indicative of lysosomal rupture. Data are shown as average +/− SD of object numbers, fluorescence intensity, area per cell, or average fluorescence intensity per identified object respectively from a representative experiment. Data were fitted with a gaussian curve for visual purposes only.

Previous research from our group showed that PS-NH_2_ NPs caused swelling of the lysosomes at concentrations between 25–100 µg/ml as part of the apoptotic process [47]. As our HCA approach showed a dose-dependent increase in the total cellular fluorescence of the lysosomes after exposure to PS-NH_2_ for 24 hours we decided to perform a detailed correlation of size and intensity of Lysotracker positive compartments in the images acquired from two representative cell lines, 1321N1 and HepG2 cells. Although this software analysis is performed on wide field epifluorescence microscopy images, it could distinguish acidified compartments as individual objectswhich might vary in size and intensity within the cell population as consequence of exposure and accumulation of NPs. As illustrated in figure 6A, the Spot Detection Bio-application identified highly acidic compartments positive for the Lysotracker green staining.

The Spot Detector analysis measured the number of identified positive fluorescent objects (spots), their intensity and area for each cell, and the intensity of individual spots. The average values of 30 images from three wells of a representative dose response experiment for 1321N1 and HepG2 cells were plotted in Fig. 6B. HepG2 cells showed a higher degree of vacuolation, but underwent a comparable dose response to 1321n1 cells. By looking at the individual graphs we observed a similar trend in the increase of spot numbers, area and intensity, but a closer observation of the different parameters integrated in the same plot revealed more dynamic inter-relationships. Within the same cell line and the same experiment the spot numbers/cell increased at the lowest concentration followed by an increase in the cellular area and lastly by an increase in the intensity/cell; similarly the spot numbers started decreasing at concentrations higher than 12.5 µg/ml, followed by a decrease in the area and intensity of the spots (Fig. 6C). These identified sequences correlate very well with the previous observations on the swelling and rupturing of lysosomes caused by PS-NH_2_ NPs.

### PS-NH_2_ NPs cause loss of lysosomal functionality through accumulation of neutral- and phospholipids

**Figure 7:**
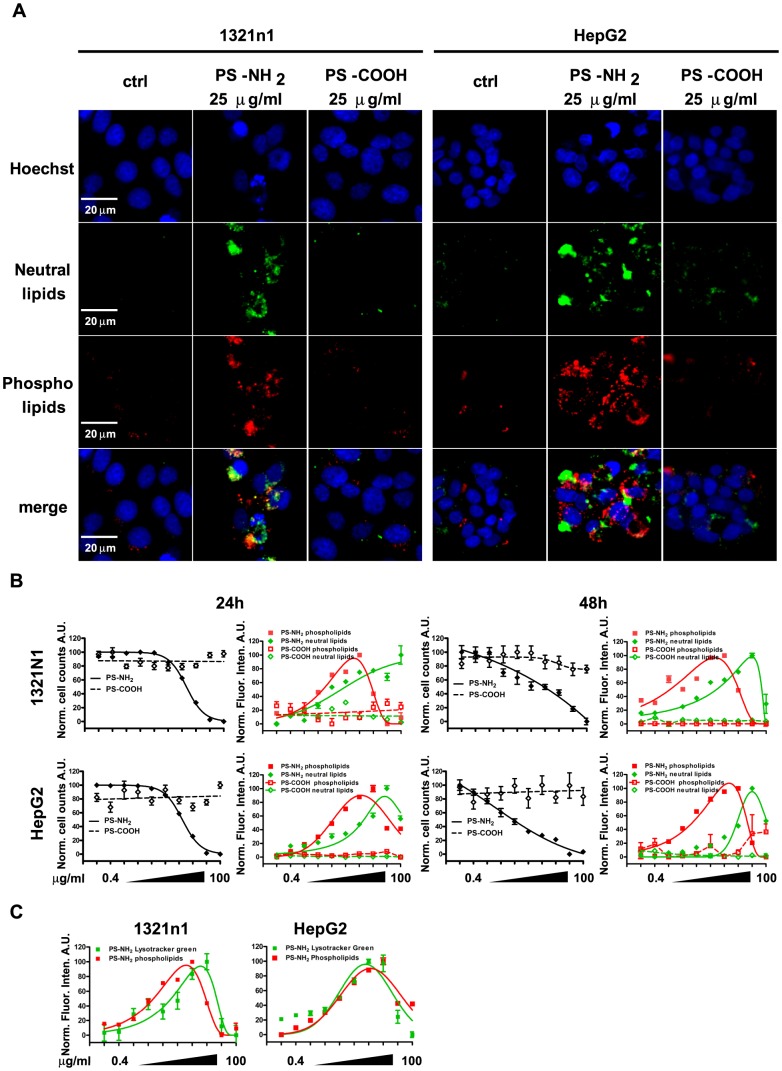
PS-NH_2_ NPs cause dose-dependent accumulation of phospholipids and neutral lipids in 1321N1 and HepG2 cells. 1321n1 and HepG2 cells were exposed to vehicle (ctrl) or increasing concentrations of PS-COOH or PS-NH_2_ NPs for 24 or 48 hours and stained with the LipidTOX phospholipidosis and steatosis detection kit (Life Technologies). A. Representative images of 1321N1 and HepG2 cells exposed to vehicle or 25 µg/ml PS-NH_2_ and PS-COOH NPs for 24 hours. PS-NH_2_ NPs caused intracellular accumulation of phospho- and neutral lipids. Scale bar = 20 µm. B. Graphs showing dose-dependent reduction in cell numbers and associated intracellular accumulation of phospho- and neutral lipids after exposure to NPs for 24 or 48 hours. Dose-dependent accumulation was observed in both cell lines after exposure to PS-NH_2_ NPs; PS-COOH NPs did not cause lipid accumulation nor altered the nmbers of cells acquired. C. Comparison of Lysotracker Green and phospholipid accumulation after exposure to increasing concentrations of PS-NH_2_ NPs for 24 hours. Significant overlap can be observed suggesting that lysosomal swelling is closely correlated with lipid accumulation caused by PS-NH_2_ NPs. Data are shown as average +/− SD of 45 acquired images from three independent experiments. Data were fitted with a gaussian curve for visual purposes only.

We have identified that increase in acidity associated with swelling of the lysosomal compartments is a common feature observed in all the cell lines investigated exposed to PS-NH_2_ NPs, and together with our previous finding it exposes a scenario where lysosomal damage is the triggering event that starts the apoptotic cascade [40]; we decided therefore to investigate the mechanisms associated with PS-NH_2_ NPs-induced lysosomal damage. It was observed already many years ago that amphyphylic cationic drugs caused lysosomal damage through accumulation of lipids inside the lysosomes of cells, a phenomenon called lipidosis [48], and later it was suggested that a specific class of cationic drugs, aminoglycosides, were able to bind polar lipids and accumulate them in the lysosomes, causing the formation of phospholipid-containing myeloid bodies, that would cause the lysosomal membrane to stretch and eventually rupture. More recently it was suggested also that cloride channels expressed on the lysosomal membrane are central in the regulation of the proton balance inside the lysosomes [49]. As these observations were very similar to the swelling and rupturing of lysosomes observed by us previously [27] and in this study, we applied the LipidTOX phospholipidosis and steatosis detection kit for High Content Screening (Life Technologies) to assess accumulation of neutral- and phospholipids in 1321N1 and HepG2 cells exposed to increasing doses of PS-NH_2_ NPs (as in previous experiments) for 24 hours and 48 hours. Representative images of cells exposed to 25 µg/ml PS-NH_2_ NPs following HCA showed that indeed there was a substantial accumulation of both neutral and phospholipids in both cell lines; moreover although the images were acquired through an epifluorescence microscope it was observed that the fluorescence of the neutral lipid stain and that of the phospholipid stain were separate, suggesting that lipids of different nature were accumulated in different compartments. Exposure to PS-COOH NPs on the contrary did not cause lipid accumulation (Fig. 7A). The analysis of the fluorescence intensity over the different doses confirmed a dose-dependent increase in accumulated neutral and phospholipids in both 1321N1 and HepG2 cells after 24 or 48 hour exposure, which correlated well with the induction of cell toxicity, as shown by the dose-dependent reduction in cell numbers per field of view (Fig. 7B). The dose-dependent increase in phospholipid and neutral lipid staining potentially reflects different engagement of the lysosomal pathway with different lipidic vescicles; the observed reduction in fluorescence staining observed after 24 or 48 hours exposure could be correlated with the dismantling of membrane structures during the apoptotic process. It is worth noting that the phospholipid staining was applied at the start of the experiment, according to manufacturer's instructions, while the neutral lipid staining was applied at the end of the experiment. The different application time of the two stainings could account for the different reduction occurring in the staining intensity, which was observed at doses higher than 25 µg/ml (phospholipid staining) or at doses higher than 50 µg/ml (neutral lipid staining). The dose response graphs also confirmed that PS-COOH NPs did not cause accumulation of lipids in the cells and did not cause lysosomal damage. Interestingly, when comparing the dose response curve of the lysotracker green and the phospholipid stain after 24 hour exposure to PS-NH_2_ NPs from independent experiments we observed a very good overlap, particularly for the HepG2 cells, suggesting that there is a tight correlation between the accumulation of intracellular phospholipids and the acidification of lysosomal compartments; this correlation could suggest that the lysosomal damage caused by PS-NH_2_ NPs could be caused by phospholipids trapped on the surface of the NPs that accumulate inside the lysosomes and generate myeloid bodies, which similarly to aminoglycosides caused swelling and rupture of the lysosomal membrane. Furthermore this observation also suggests that lysotracker green could be used as a predictive marker for accumulation of phospholipids inside lysosomes caused by cationic NPs.

### Human primary astrocytes show a very similar response to 1321N1 astrocytoma cells after exposure to PS-NH_2_ NPs

**Figure 8:**
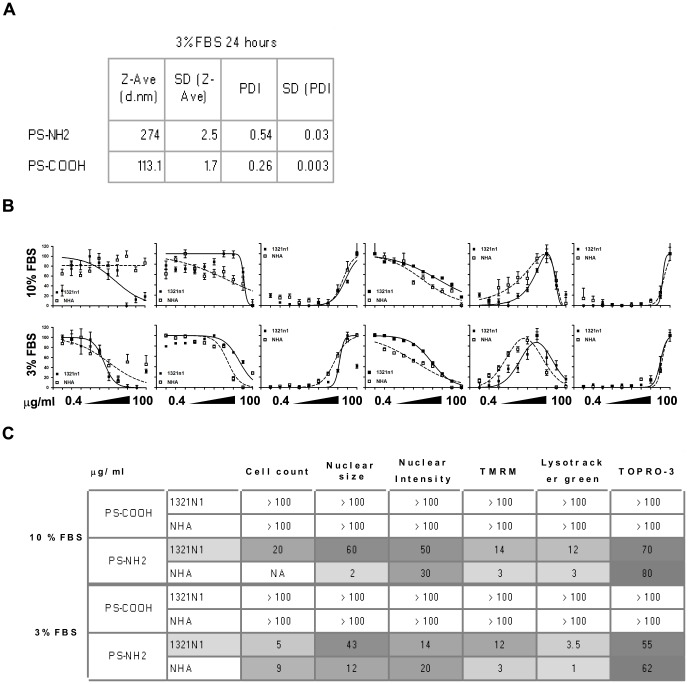
Primary human astrocytes respond to PS-NH_2_ and PS-COOH comparably to 1321N1 astrocytoma cells. 1321N1 and NHA cells were exposed to vehicle (ctrl) or increasing concentrations of PS-COOH or PS-NH_2_ NPs for 24 hours and analysed using HCA. Comparison of results obtained between the two cell lines in DMEM supplemented with 10% FBS (A) or with 3% FBS (B). In the graphs 1321n1 cells are represented by the continuous line (▪) while HNA cells are represented by the dashed line (□) C. EC50/IC50 measurements for both cell lines exposed to NPs in presence of 10% or 3% FBS. NHA primary astrocytes showed slightly higher sensitivity than 1321N1 astrocytoma cells, with EC50/IC50 values in the same order of magnitude. Data are shown as average +/− SD of 30 acquired images from a representative experiment. Curve fitting and EC50/IC50 were obtained as described in the Methods. Fluo-4 and Lysotracker green dose response plots were fitted with a gaussian curve for visual purposes only.”

Cancer-derived cell lines are widely used as *in vitro* predictive models of human toxicity; these cell lines though might carry genetic mutations in survival/apoptosis cellular pathways that may alter the response to toxic agents, including potentially NPs [50]–[54]; in order to assess this possibility we compared human 1321N1 astrocytoma cells with human NHA primary astrocytes exposed for 24 hours to the same doses of PS-COOH and PS-NH_2_ NPs; since the culturing conditions for primary astrocytes are different than the cell lines previously investigated, i.e. cells are grown in medium supplemented with 3% FBS, we performed DLS measurements to assess size distribution of PS-COOH and PS-NH_2_ NPs in medium supplemented with 3% FBS and exposed both cell lines to these NPs for 24 hours in medium supplemented with 3% FBS. To allow us to compare the results with our previous observations we also exposed both cell lines to NPs in medium supplemented with 10% FBS. DLS size distribution measurements showed that both NPs are increasingly agglomerated once dispersed in cell culture medium supplemented with 3% FBS (Fig. 8A, Figure S8 in File S1), suggesting that the ratio between proteins and NPs is not sufficient to fully stabilise the surface of NPs; HCA performed on both cell types showed that exposure in medium supplemented with 3% FBS generated dose-response curves at lower concentrations compared to exposure in medium supplemented with 10% FBS, and in both experimental conditions NHA astrocytes were slightly more sensitive than 1321N1 astrocytoma cells (Fig. 8B). Analysis of the EC50/IC50 highlighted that in both experimental conditions Lysotracker green gave the lowest EC50 for both cell lines and the same sequence of EC50/IC50 thresholds was identified as previously observed. Moreover NHA cells displayed EC50/IC50 values that were lower than what observed in 1321N1 cells, although still in the same fold range (Fig. 8C). These data suggest that once established that 1321N1 astrocytoma cells are slightly more resistant than their primary counterpart, however both cell lines could be used as a reliable cell model to predict the response of glial cells to NPs' exposure.

## Discussion

Amine modified polystyrene (PS-NH_2_) NPs are well characterised NPs both in terms of their physico-chemical characteristics in biological fluids and their induction of apoptosis in cultured cells; they are therefore a good candidate as positive control nanoparticle for apoptosis and for the implementation and validation of *in vitro* testing platforms.

This is the first comprehensive study where several cell lines representative of different organs in the body were compared for their response to PS-NH_2_ NPs, using a High Content Analysis appraoch that measures several parameters of cellular toxicity simultaneously.

The comparison of different cell lines exposed to the same amine-modified polystyrene NPs in the same experimental conditions showed that with the exception of Raw 264.7 all the other cell lines investigated had the same sequence of EC50/IC50 thresholds, starting with lysosomal acidification, followed by mitochondrial depolarisation, and simultaneous nuclear condensation, cytosolic calcium increase and plasma membrane permeabilization. The identified sequence is consistent with apoptotic cell death and confirms previous observations that amine-modified polystyrene NPs induce apoptosis in 1321N1 cells [40]. On the contrary for Raw264.7 cells we observed that the EC50/IC50 thresholds were very close, moreover the nuclear morphological changes observed differ from the other cell lines investigated. While nuclear condensation was observed generally in all the cell lines investigated, Raw264.7 cells exposed to increasing concentrations of PS-NH_2_ NPs showed dose-dependent nuclear swelling, which is indicative of a different mechanism of cell death, i.e. necrosis. It was indeed demonstrated recently by a very comprehensive study that human macrophages exposed to PS-NH_2_ NPs underwent necrotic cell death [55].

The similar thresholds observed for cytosolic calcium increase and plasma membrane permeabilization suggests that the observed increased cytosolic calcium was a late event in the apoptotic cascade and was potentially caused by influx of extracellular calcium through the depolarised/permeabilized plasma membrane. Further investigation is required to analyse specific intracellular calcium signalling connected to the cell death pathway.

The different cell death outcomes observed in Raw264.7 and the other cell lines investigated are indeed intriguing and raise the question on the biological variables that control them; we believe that such differences could be explained by diverse cell uptake rates of the amine-modified NPs. The Raw264.7 macrophages are by definition scavenger cells programmed to segregate foreign bodies therefore activating the primary immune response; moreover in a previous publication [56], our group showed that Raw264.7 cells display higher uptake rates of carboxyl- modified polystyrene NPs.

The parameter identified with lower EC50 was Lysotracker green, an acidotrophic fluorophore which accumulates in acidic compartments. This suggests that alteration of the lysosomes could be the factor that triggers the apoptotic/necrotic response, which is consistent with our previous findings that NPs accumulate in the lysosomes as their final destination in the cells [21], [27] and with a time resolved study showing that indeed acidification of the lysosomes is the first event triggered in the apoptotic pathway activated by PS-NH_2_ NPs [47]. These finding show how the series of EC50 thresholds could be predictive of temporal sequences of events in the apoptotic cascade; by comparing the Lysotracker green EC50 thresholds of Raw264.7 and the other cell lines exposed to PS-NH_2_ NPs we could also observe that the EC50 is significantly lower for Raw267.4 cells, supporting further the hypothesis that these cells may have higher uptake rates compared to the other cell lines investigated and therefore suggesting a thresholding effect where a higher uptake rate can cause a switch between apoptotic to necrotic cell death. This phenomenon has been well characterised for chemically-induced cell death where molecular mediators such as ATP or nitric oxide have been identified as responsible for the switch [57]; also glutamate excitotoxicity has been reported to activate apoptosis or necrosis in primary neurons using differential activation of calcium-mediated pathways [58], [59].

The cellular and molecular pathways associated with acidification of lysosomes following exposure to PSNH_2_ NPs have not been clarified yet and many theories have been formulated. It was shown already in the 1970's that cationic drugs caused accumulation of polar lipids in the lysosomes [48], [60]; in the case of aminoglycosides, for example, their accumulation in the lysosomes and their interaction with phospholipids would lead to accumulation of phospholipid-containing myeloid bodies that cause the lysosomal membranes to stretch and eventually rupture [61]. By measuring accumulation of neutral and phospholipids in cells exposed to PSNH_2_ NPs we demonstrated that indeed the accumulation of PS-NH_2_ NPs in lysosomes not only activated apoptotic or necrotic cell death but it also caused impaired lysosomal functionality. Potentially similarly to aminoglycosides PS-NH_2_ NPs could cause, among other phenomenons, accumulation of myeloid bodies that ultimately would be responsible for the rupture of the lysosomal membranes and release of lysosomal contents.

A current theory to explain the effect of cationic NPs on lysosomal physiology suggests that once cationic NPs accumulate in the lysososmes they cause an influx of H^+^, which could be followed by accumulation of water by osmosis, therefore also resulting in swelling and eventual rupture of the lysosomal membrane due to hosmotic shock; the mechanisms proposed to explain this phenomenon include the proton sponge effect, i.e. the buffering potential of the surface of NPs could lead to excessive influx of protons in the lysosomes, although the current literature suggests that this mechanism could be cell type dependent [26].

Interestingly it was recently reported that the cloride channel ClC-7 on the lysosomal membrane is a Cl^−^/H^+^ exchanger [49]; this ion transporter exchanges Cl^−^ ions with H^+^ as a buffering mechanism for the pH of the lysosomal environment. In light of this we could hypotesize that inbalances in the receptor activity generated by the positive surfaces of accumulated NPs may cause excessive influx of H^+^ in the lysosomes; such theory also provides a good correlation with the experimental evidence shown in this manuscript and previous publications from our group and supports impairement in functionality of lysosomes in cells exposed to lethal concentrations of PS-NH_2_ NPs, which may results in impaired recycling of endosomal vescicles and subsequent accumulation of lipidic structures resulting from the fusion of endosomes and lysosomes.

This novel finding, together with the good overlap observed between the Lysotracker green and the phospholipid accumulation dose response curves suggests a good correlation between acidification of the lysosomes measured using lysotracker green and impaired lysosomal functionality caused by accumulation of neutral and phospholipids.

Our HCA platform demonstrated the power to resolve different cell death pathways according to differences in nuclear morphology and different threshold sequences of EC50/IC50 of the analysed parameters, adding to the screening approach a mechanistic interpretation of the cellular pathways involved; the nuclear swelling observed in Raw264.7 cells opposed to apoptotic nuclear condensation observed in the other cell lines, together with the absence of EC50 threshold sequences for the parameters analysed suggests a sudden non-regulated cell death, consistent with necrosis, as recently observed by Lunov O. [55]. The different cell death pathways activated following exposure to amine-modified polystyrene NPs are currently being investigated.

Cancer-derived cell lines are known to carry genetic mutations that may affect their response to cytotoxic stimuli, mainly associated with defects in the apoptotic pathways, or over-activation of survival pathways [50]–[54]. In light of this we were interested to compare the cell lines tested with the equivalent primary cell model. We compared the 1321n1 astrocytoma cells with NHA primary human astrocytes exposed to PS-NH_2_ and PS-COOH NPs; since 1321n1 and NHA cells are grown in different culturing conditions we tested both cell lines in both culturing conditions. Interestingly we observed that the two cell lines showed very comparable dose response curves to PS-NH_2_ NPs in both culturing conditions, suggesting that 1321n1 cells show an apoptotic response that is comparable to primary human astrocytes in the specific culturing conditions tested and for the parameters investigated, and they could be considered as a reliable model for investigation of toxicity induced by NPs.

Overall the HCA platform showed comparable sensitivity to OECD tests such as the MTS or PI uptake, with the advantage of measuring several parameters simultaneously while requiring little manipulation of the samples; HCA could therefore be a very powerful tool to accelerate safety testing and keep the pace with the many engineered nanomaterials generated, in compliance with OECD guidelines. Another advantage of the HCA platform is that it is independent from any particular cell type and it could therefore be implemented using advanced *in vitro* models such as cell monolayers representing biological barriers, co-cultures or even three-dimensional models representing organ-like structures; such advanced models, paired with the appropriate biological fluid could exploit the full analytical and screening potential of HCA for a realistic safe assessment performed *in vitro*, reducing greatly the need for animal testing.

### Conclusions

Understanding the cellular pathways activated by nanomaterials once they get in contact with biological systems and implementation of high throughput *in vitro* methods for safety assessment of nanomaterials are key factors to accelerate development of nanotechnology, both from the scientific point of view of determining the fate of nanoparticles in biological organisms, and further build accurate predictive models to simulate such processes, but also for the aspect of promoting safer use of nanotechnology in commercial products.

With this study we have implemented and validated an *in vitro* High Content Analysis platform that lays the foundations to achieve such goals. Mechanistic data collected using HCA in a rapid and reproducible format has the potential to feed into system biology predictive models and accelerate access of nanomaterials to the market.

## Supporting Information

File S1 **Supporting figures.**
(DOCX)
