# Supplementary material for: High Content Analysis Provides Mechanistic Insights on the Pathways of Toxicity Induced by Amine-Modified Polystyrene Nanoparticles
Source: PLoS One. 2014 Sep 19;9(9):e108025. doi: 10.1371/journal.pone.0108025 (PMC4169620; doi:10.1371/journal.pone.0108025)
Supplement: File S1 — Supporting figures. (DOCX) [file pone.0108025.s001.docx]

Supporting Information

High Content Analysis provides mechanistic insights on the pathways of toxicity induced by amine-modified polystyrene nanoparticles.

*Sergio Anguissola^*^, David Garry , Anna Salvati , Peter J. O'Brien , Kenneth A. Dawson ^*^.*

**Figure S1**


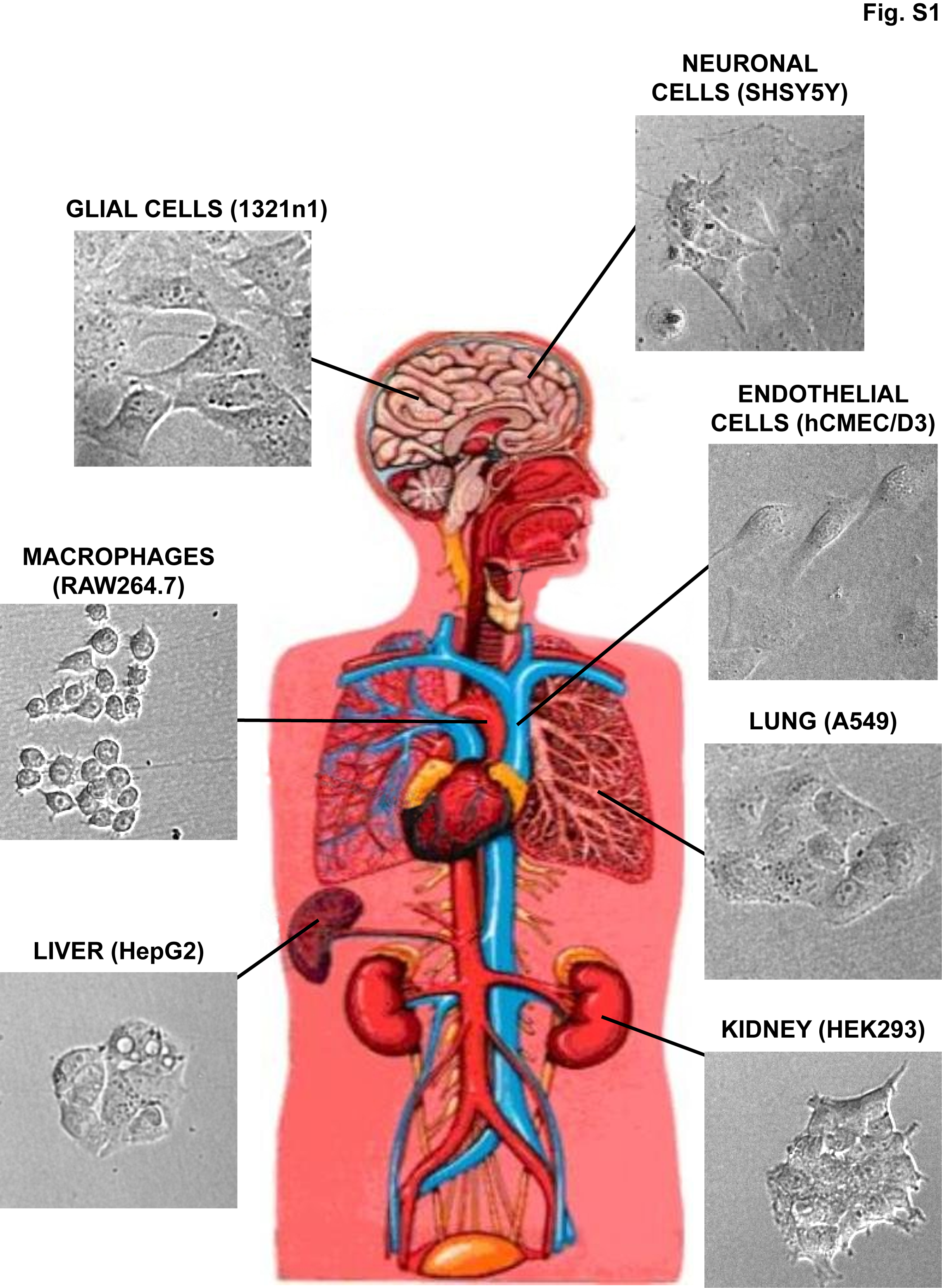


**Figure S1. Panel of selected cell lines representing the main routes of exposure, accumulation and clearance from the body.**

1321N1, SHSY5Y, A549, hCMEC/D3, HEK293, HepG2 and RAW264.7 cells were used as models for exposure, accumulation and clearance routes from the body. Brightfield images of the indicated cell types are shown.

**Figure S2**

**
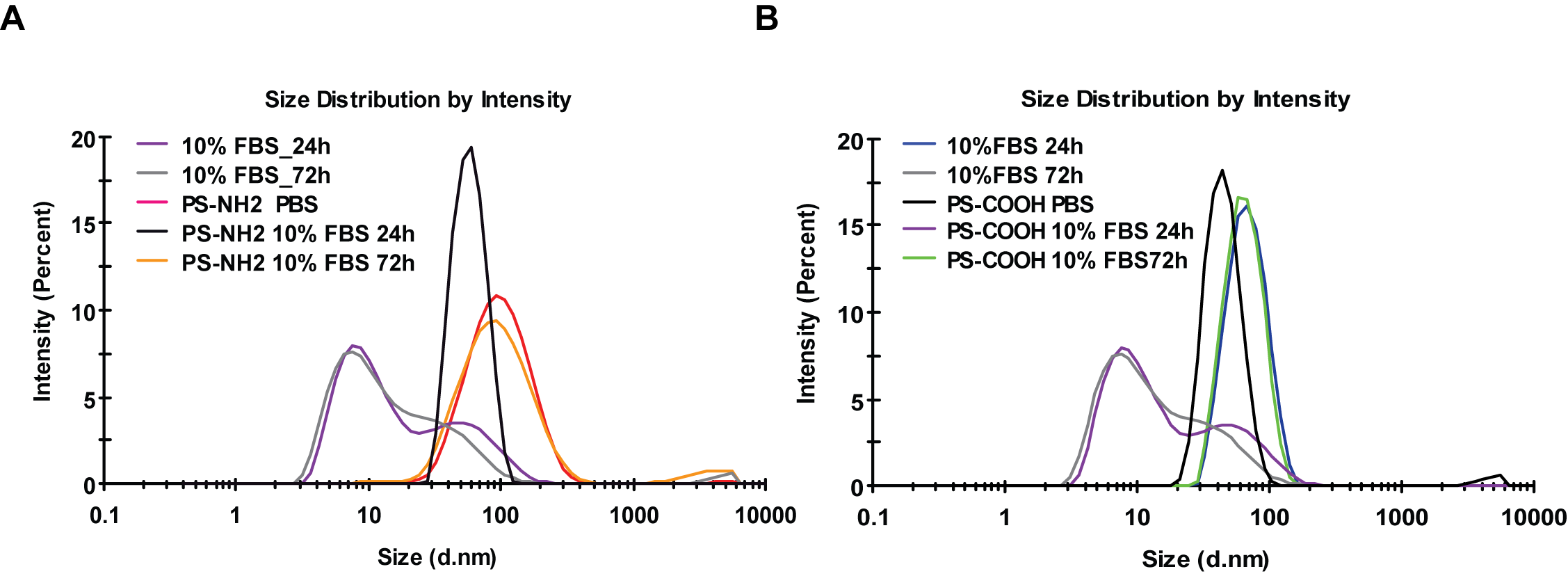
**

**Figure S2. DLS characterisation of the nanoparticle dispersions.**

DLS measurements of nanoparticles dispersed in cell culture medium supplemented with 10% FBS for 24 hours or 72 hours were processed using the CONTIN analysis. A) represents PS-NH_2_ NPs and B) shows PS-COOH NPs at the indicated times.

The dispersions were incubated for the indicated times at 37°C and 5% CO2 to mimic the exposure conditions to cells. Compared to the dilutions in phosphate buffer (PBS), dispersions of nanoparticles in cell culture medium resulted in increased size, prevalently due to formation of the protein corona (c.f. black and coloured curves).

Further, only minor changes of the main size distribution peak could be observed between the two time points, suggesting that no significant agglomeration occurred over the duration of the experiment. For reference, the profile of protein aggregates from the cell culture medium alone can be observed (gray and black lines). Data are shown as average of 3 replicate measurements.

**Figure S3**

**
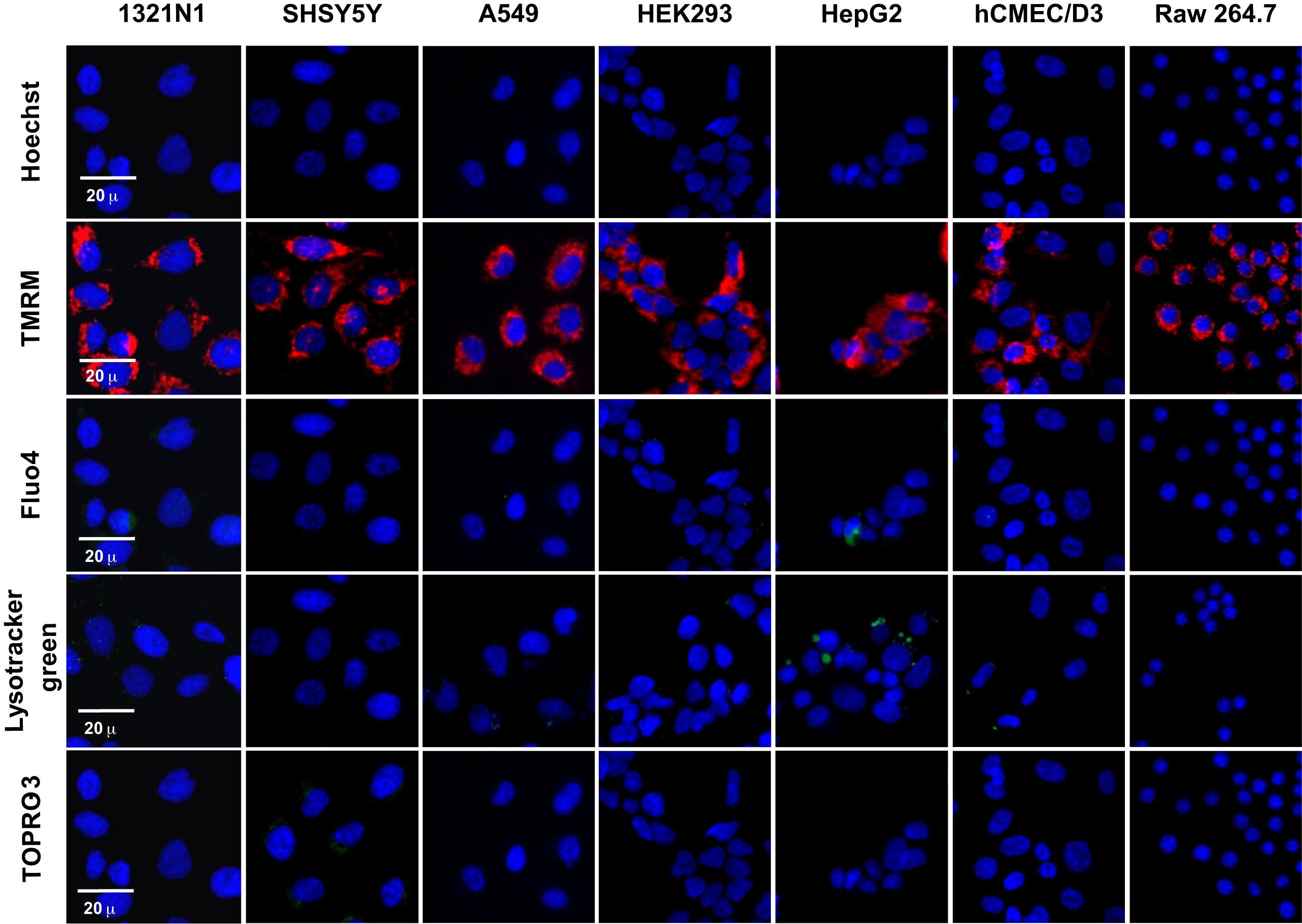
**

**Figure S3.**

All the cell lines investigated, with the experimental settings utilised for HCA, showed polarised mitochondria (TMRM) and undetectable levels of calcium signalling (Fluo4), lysosomal acidification (Lysotracker Green) and plasma membrane permeabilisation (TOPRO-3) under standard culturing conditions. HepG2 cells showed basal lysosomal activity which was greatly enhanced after exposure to PS-NH_2_ NPs (Figure 2, 3).

The indicated cell lines were exposed to vehicle (ctrl) for 24 hours; images were acquired using HCA to assess changes in nuclear morphology (Hoechst), mitochondrial membrane potential (TMRM), cytosolic calcium levels (Fluo-4), lysosomal acidification (Lysotracker green), and plasma membrane integrity ( TOPRO-3); representative images show 1321N1, SHSY5Y, A549, RAW264.7, hCMEC/D3, HepG2, HEK293 cells exposed to vehicle (ctrl). Scale bar = 20 μm.

**Figure S4**

**Figure S4. PS-NH_2_ NPs induced different cell death pathways in A549 or RAW264.7 cells.**

A549 cells exposed to PS-NH_2_ NPs showed a population of early apoptotic cells (AnnexinV positive/PI negative), prominent at the exposed dose of 50 μg/ml and indicative of apoptotic progression, while RAW264.7 cells revealed a prominent population of necrotic cells (AnnexinV positive/PI positive) already at the exposed dose of 25 μg/ml, suggesting that Ps-NH_2_ NPs caused necrosis in RAW264.7 cells.

A549 (A) and RAW264.7 (B) cells were exposed to PS-NH_2_ NPs at the indicated concentrations for 24 hours and processed for flow cytometry. AnnexinV-FITC staining showed exposure of phosphatydilserine on the plasma membrane, while Propidium Iodide (PI) revealed loss of plasma membrane integrity. Data are shown as average +/- SD of 3 replicates from representative experiments.

**Figure S5**

| **72 h** | | | | | | | | |
| --- | --- | --- | --- | --- | --- | --- | --- | --- |
|  | **mg/ml** | **Cell count** | **Nuclear size** | **Nuclear Intensity** | **TMRM** | **Fluo-4** | **Lysotracker green** | **TOPRO-3** |
| **PS-COOH** | 1321N1 | > 100 | > 100 | > 100 | > 100 | > 100 | > 100 | > 100 |
|  | SHSY5Y | > 100 | > 100 | > 100 | > 100 | > 100 | > 100 | > 100 |
|  | RAW264.7 | > 100 | > 100 | > 100 | > 100 | > 100 | > 100 | > 100 |
|  | A549 | > 100 | > 100 | > 100 | > 100 | > 100 | > 100 | > 100 |
|  | hCMEC | > 100 | > 100 | > 100 | > 100 | > 100 | > 100 | > 100 |
|  | HepG2 | > 100 | > 100 | > 100 | > 100 | > 100 | > 100 | > 100 |
|  | HEK293 | > 100 | > 100 | > 100 | > 100 | > 100 | > 100 | > 100 |
| **PS plain** | 1321N1 | > 100 | > 100 | > 100 | > 100 | > 100 | > 100 | > 100 |
|  | SHSY5Y | > 100 | > 100 | > 100 | > 100 | > 100 | > 100 | > 100 |
|  | RAW264.7 | > 100 | > 100 | > 100 | > 100 | > 100 | > 100 | > 100 |
|  | A549 | > 100 | > 100 | > 100 | > 100 | > 100 | > 100 | > 100 |
|  | hCMEC | > 100 | > 100 | > 100 | > 100 | > 100 | > 100 | > 100 |
|  | HepG2 | > 100 | > 100 | > 100 | > 100 | > 100 | > 100 | > 100 |
|  | HEK293 | > 100 | > 100 | > 100 | > 100 | > 100 | > 100 | > 100 |
| **PS-NH2** | 1321N1 | 5.8 | 40 | 10 | 2.8 | 10 | 2 | 18 |
|  | SHSY5Y | 7 | 50 | 20 | 2 | 14 | 3 | 39 |
|  | RAW264.7 | <0.4 | 1.6 | 3.4 | <0.4 | 2.5 | 1.3 | 11 |
|  | A549 | 2.8 | 28 | 19 | 4 | 34 | 2.3 | 30 |
|  | hCMEC | 5 | 20 | 14 | 13 | 5 | 3 | 15.3 |
|  | HepG2 | 5 | 60 | 2 | 13 | 26 | 3 | 3.3 |
|  | HEK293 | 6.6 | 8 | 7 | 6.6 | 2.4 | 2.9 | 2.3 |

**Figure S5. Sequences of EC50 thresholds suggest that all cell lines tested except RAW264.7 execute apoptotic cell death, while RAW264.7 undergo a non-regulated form of cell death.**

EC_50_/IC_50_ for each parameter measured for the selected cell lines exposed to vehicle (ctrl) or increasing doses of PS-Plain, PS-COOH or PS-NH_2_ NPs, for 24 hours or 72 hours were calculated as described in the Methods. EC50 / IC50 measured values after exposure for 72 hours are shown.

**Figure S6**


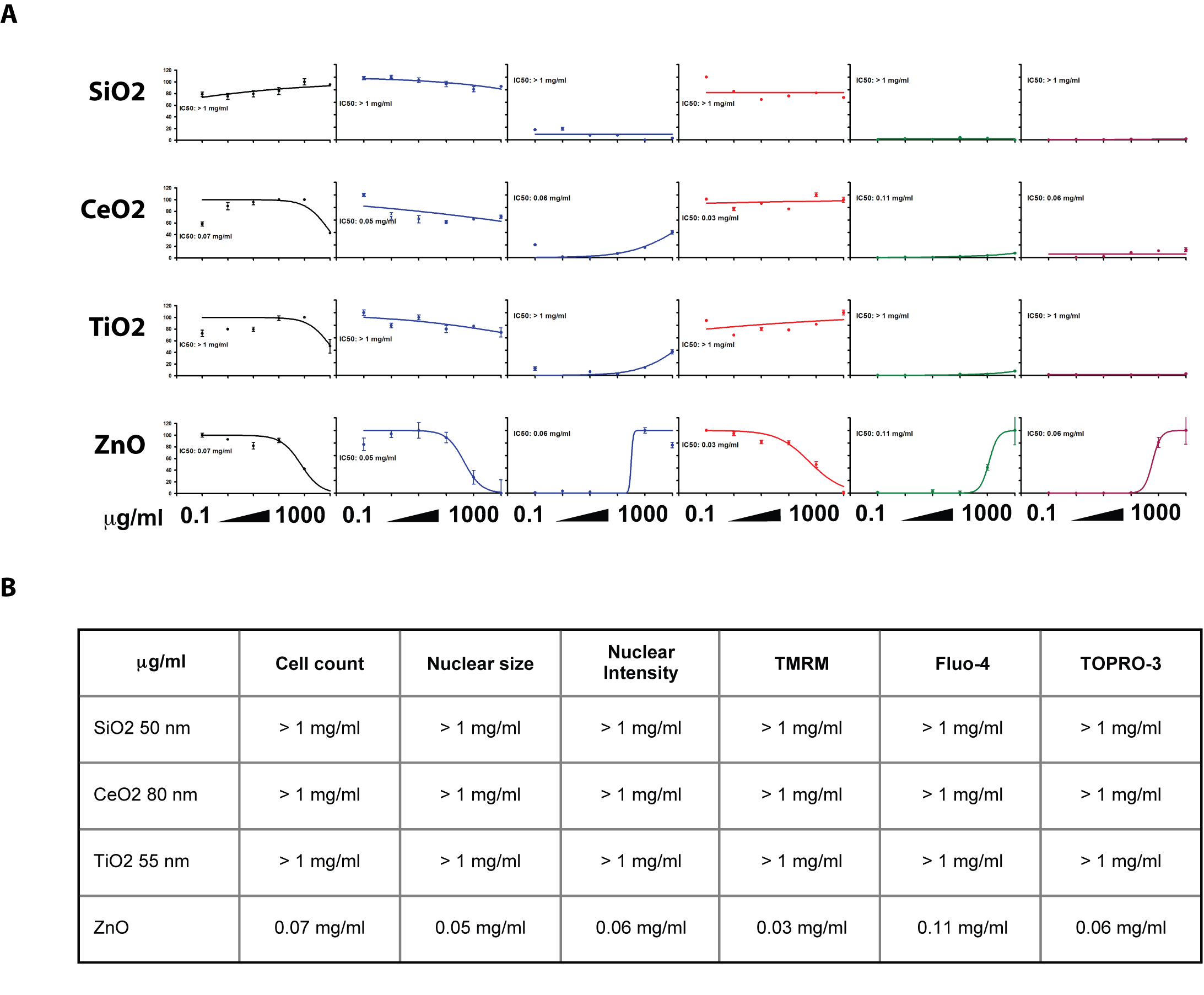


**Figure S6. The HCA platform is applicable to a wide range of nanoparticles.**

A broad range of nanoparticles were tested using the HCA platform, exposed to 1321n1 cells in a broad range of concentrations from 0.1 μg/ml to 1 mg/ml over a period of 72 hours. For descriptive purposes the examples included were nanopartcles with well characterised dispersion properties in cell culture medium, 50 nm SiO_2_ [[1](#_ENREF_1)], 180 nm CeO_2_, 55 nm TiO_2_ [[2](#_ENREF_2)] and 100 nm ZnO NPs. Apart from ZnO NPs, which caused significant levels of toxicity, all the other NPs shown were very well tolerated even at very high concentrations.

1321n1 cells were exposed to vehicle (ctrl) or increasing concentration of nanoparticles for 72 hours. HCA was employed to assess changes in nuclear morphology (Hoechst), mitochondrial membrane potential (TMRM), cytosolic calcium levels (Fluo-4), lysosomal acidification (Lysotracker green), and plasma membrane integrity ( TOPRO-3);

The graphs show for each parameter the dose-dependent response to applied NPs.

Data are shown as average +/- SD of 5 acquired images from a representative experiment.

**Figure S7**

**
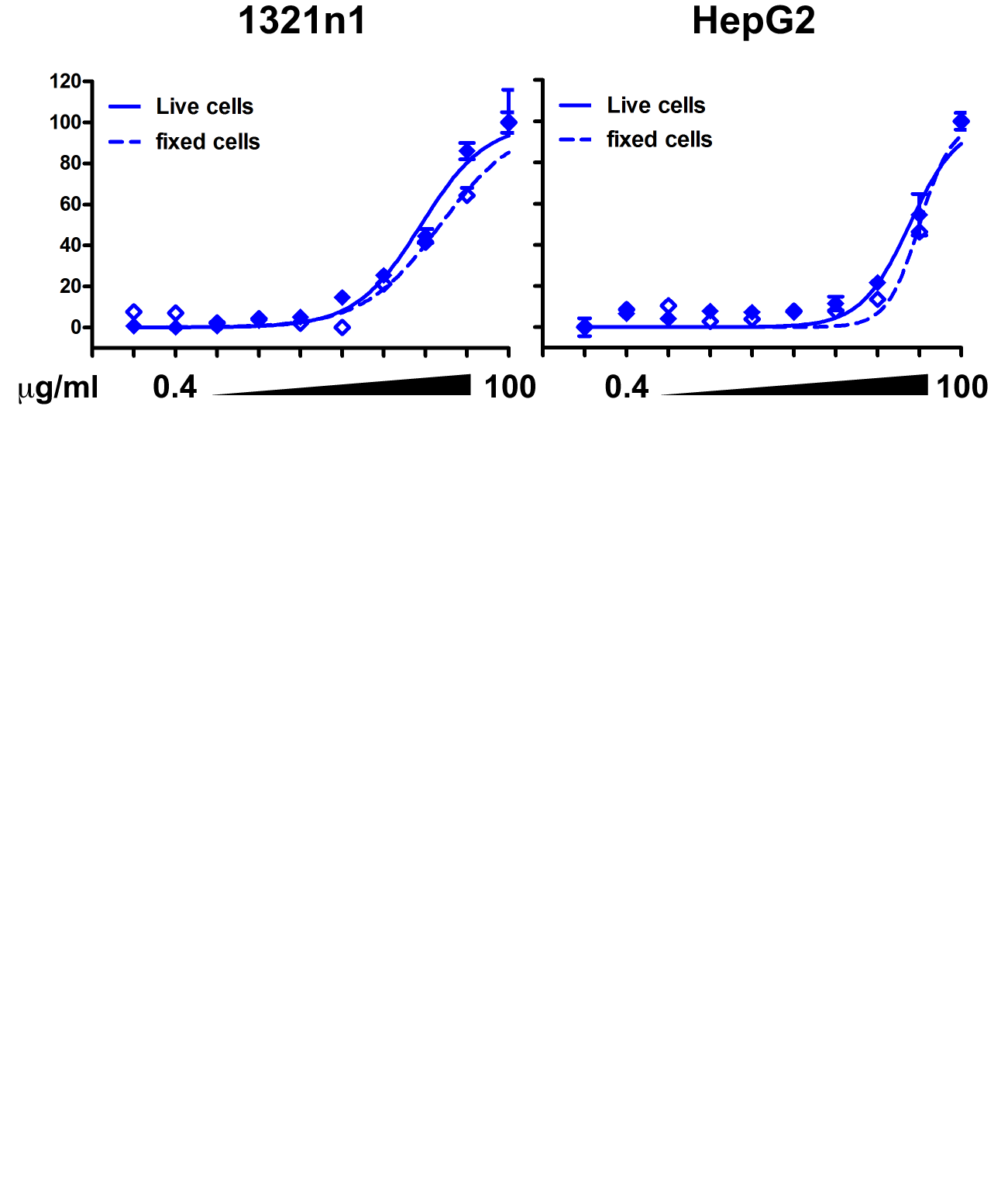
**

**Figure S7. The HCA platform translates effectively to applications requiring fixed cells.**

Comparable changes in nuclear intensity were observed in both live or fixed cells exposed to increasing doses of PS-NH_2_ NPs for 24 hours.

1321n1 and HepG2 cells were exposed to vehicle (ctrl) or increasing concentration of PS-NH_2_ nanoparticles for 24 hours. HCA was employed to assess changes in nuclear intensity (Hoechst);

The graphs show the dose-dependent response to applied NPs.

Data are shown as average +/- SD of 30 acquired images from representative experiments.

**Figure S8.**

**
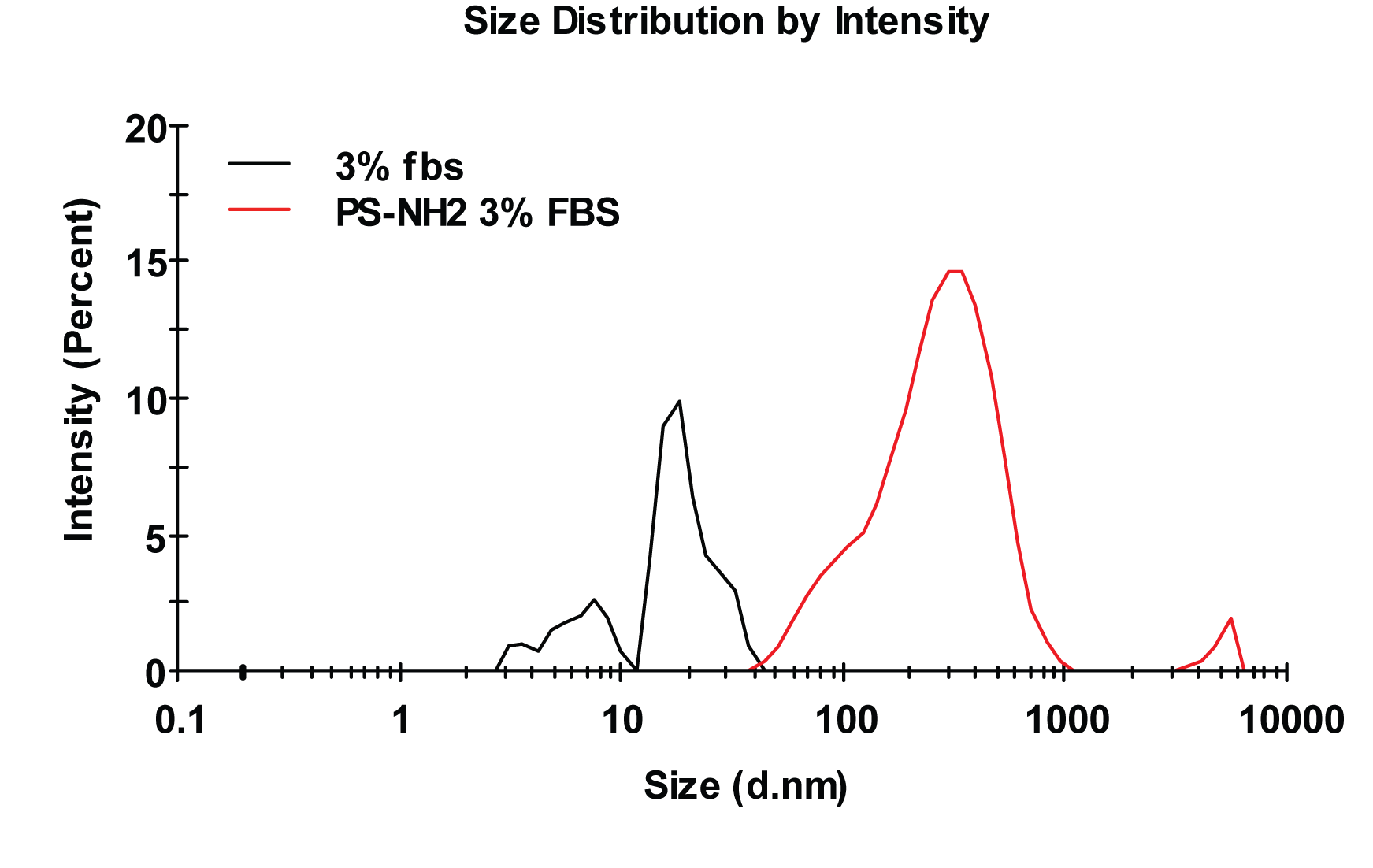
**

**Figure S8. DLS characterisation of the nanoparticle dispersions in cell culture medium supplemented with 3% FBS.**

DLS measurements of PS-NH_2_ NPs nanoparticles dispersed in cell culture medium supplemented with 3% FBS for 24 hours were processed using the CONTIN analysis.

The dispersions were incubated for the indicated times at 37°C and 5% CO2 to mimic the exposure conditions to cells. Compared to the dilutions in phosphate buffer (PBS), dispersions of nanoparticles in cell culture medium supplemented with 3% FBS resulted significantly agglomerated.

**Refeerences**

[1] Xiao R, Power RF, Mallonee D, Crowdus C, Brennan KM, Ao T, et al. A comparative transcriptomic study of vitamin E and an algae-based antioxidant as antioxidative agents: investigation of replacing vitamin E with the algae-based antioxidant in broiler diets. Poultry science. 2011;90:136-46.

[2] Ramirez-Garcia S, Chen L, Morris MA, Dawson KA. A new methodology for studying nanoparticle interactions in biological systems: dispersing titania in biocompatible media using chemical stabilisers. Nanoscale. 2011;3:4617-24.
